# Predictive Maintenance and Fault Detection for Motor Drive Control Systems in Industrial Robots Using CNN-RNN-Based Observers

**DOI:** 10.3390/s25010025

**Published:** 2024-12-24

**Authors:** Chanthol Eang, Seungjae Lee

**Affiliations:** Department of Computer Science and Engineering, Intelligent Robot Research Institute, Sun Moon University, Asan 31460, Republic of Korea; ngchanthol1@gmail.com

**Keywords:** fault detection, CNN-LSTM, CNN-RNN, DC motor drives, industrial robots

## Abstract

This research work presents an integrated method leveraging Convolutional Neural Networks and Recurrent Neural Networks (CNN-RNN) to enhance the accuracy of predictive maintenance and fault detection in DC motor drives of industrial robots. We propose a new hybrid deep learning framework that combines CNNs with RNNs to improve the accuracy of fault prediction that may occur on a DC motor drive during task processing. The CNN-RNN model determines the optimal maintenance strategy based on data collected from sensors, such as air temperature, process temperature, rotational speed, and so forth. The proposed AI model has the capacity to make highly accurate predictions and detect faults in DC motor drives, thus helping to ensure timely maintenance and reduce operational breakdowns. As a result, comparative analysis reveals that the proposed framework can achieve higher accuracy than the current existing method of combining CNN with Long Short-Term Memory networks (CNN-LSTM) as well as other CNNs, LSTMs, and traditional methods. The proposed CNN-RNN model can provide early fault detection for motor drives of industrial robots with a simpler architecture and lower complexity of the model compared to CNN-LSTM methods, which can enable the model to process faster than CNN-LSTM. It effectively extracts dynamic features and processes sequential data, achieving superior accuracy and precision in fault diagnosis, which can make it a practical and efficient solution for real-time fault detection in motor drive control systems of industrial robots.

## 1. Introduction

Industrial robots are an indispensable part of modern manufacturing, where they are instrumental in improving the efficiency, precision, and scalability in production processes. As manufacturing demands grow, these robots are increasingly being called upon to handle complex tasks that require consistent and accurate performance. The motor drive control system is a fundamental component that ensures precise and responsive motion control, directly impacting the quality and reliability of robotic operations and the entire manufacturing workflow. These systems are complicated, so they can have problems, especially with the motor drive control subsystem. If these issues are not resolved, they could have a significant impact on production. Fault detection and diagnosis (FDD) systems are useful because they find problems early, which helps with maintenance. Effective FDD systems not only facilitate the prompt detection and diagnosis of faults but also prevent potential downtimes, which can be costly and time consuming to fix. By reducing the need for unplanned maintenance and repairs, FDD systems contribute to a more resilient and efficient production environment. Furthermore, with the real-time monitoring of motor drive control system parameters, manufacturers can implement predictive maintenance practices, addressing faults before they cause damage. This approach accelerates troubleshooting, optimizes operational uptime, and enhances the reliability and longevity of industrial robots, ensuring they meet manufacturing standards and demands continuously. A sophisticated diagnostic system was designed to identify and analyze common faults in industrial robot joints, particularly focusing on issues like gear tooth wear. This system utilizes advanced sensors and machine learning algorithms to detect early signs of wear, misalignment, and other mechanical failures. By continuously monitoring the robot’s joints, it provides real-time feedback and predictive maintenance alerts, allowing for proactive interventions to prevent downtime, improve performance, and extend the lifespan of the robotic system. The system aims to optimize the reliability and efficiency of industrial robots, ensuring they operate smoothly in high-demand environments [1,2,3,4]. Many researchers have studied fault detection and identification in the motor drive control systems of industrial robots [5,6]. The primary objective of these studies is to identify potential failures that could occur in these systems. Some of the common failures include overstrain failure, heat dissipation failure, tool wear failure, and power failure. Detecting these failures early is critical to ensuring the continued performance, safety, and reliability of industrial robots, as these issues can lead to system breakdowns, reduced efficiency, or even complete operational failure. These failures often result from a variety of factors, such as excessive load, inadequate cooling, wear and tear of tools, or issues with power supply, all of which can negatively impact the functionality of the motor drive system. Therefore, advanced diagnostic techniques and predictive maintenance strategies are essential for mitigating these risks and improving the longevity of robotic systems [7,8,9,10,11]. Some researchers explore advanced methods for improving fault diagnosis and safety control in industrial systems. The first two papers focus on data-driven techniques for fault detection in imbalanced datasets and small sample sizes, addressing challenges like missing fault samples in systems such as traction motor bearings. They emphasize the use of both real-world and simulated data to enhance the accuracy and robustness of fault diagnosis models. The third paper discusses safety control for industrial robots, proposing a distributed distance sensor network to monitor the robot’s environment and ensure safe operation in dynamic settings. Together, these studies highlight the importance of integrating machine learning, data augmentation, and sensor-based systems to enhance the performance, reliability, and safety of industrial machinery and robots [12,13,14]. Some of the existing models, which integrate different machine learning techniques like convolutional and recurrent neural networks, are trained to identify anomalies in the signals, providing a reliable and automated way to monitor the health of insulators. The approach aims to improve the efficiency of fault detection and enable predictive maintenance, ensuring the reliability of electrical infrastructure [15].

There are many methods for enhancing the accuracy and robustness of fault diagnosis on motor drive control of robots by integrating with various deep learning models. However, in this work, we integrate the Convolutional Neural Network (CNN) model with the Recurrent Neural Network (RNN) model, which represents our primary proposed model in this paper. The combination of the two models results in an enhancement in fault detection and diagnosis due to the complementary strengths of both models. CNN can be highly effective in fault detection for motor drive control systems in robots by leveraging their ability to detect patterns and anomalies in sensor data, such as vibrations, current, voltage, and temperature signals, while RNN can significantly enhance fault detection in the DC motor drives of industrial robots, particularly by leveraging their ability to process sequential and time-dependent data. Integrating CNNs and RNNs for fault detection in the motor drive control systems of robotics combines the strengths of both models, providing a more robust and accurate approach with the following benefits:

- Enhanced feature extraction and temporal learning: CNN excels at automatically extracting spatial and temporal features from raw sensor data, while RNN tracks how faults evolve over time and identifies gradual changes or trends that may indicate emerging issues.
- Improved fault detection accuracy: The integration enables the system to detect both immediate anomalies and subtle, evolving faults, improving the overall accuracy of the fault detection system.
- Real-time detection with contextual awareness: CNN can process sensor data in real time, identifying immediate faults, while RNN provides contextual awareness by using past time steps, helping the model understand event sequences, which is crucial in robotics where faults develop over time.
- Enhances predictive maintenance: The combined model enhances predictive maintenance by not only detecting faults early but also predicting errors, which can help in planning maintenance before critical failures happen.
- CNN can recognize complex patterns that traditional models are hard to identify, and RNN can capture how these faults evolve and how mechanical wear affects performance over time.

The main contribution of this paper is to accurately predict the errors that can occur in the motor drives of industrial robots so that we can avoid breakdowns that may damage the robots and pose a danger to nearby humans. So, we developed a hybrid model combining CNNs and RNNs that improves predictive maintenance and fault detection in industrial robots by combining CNN’s feature extraction capabilities with RNN’s efficiency in analyzing short-term temporal dependencies. CNN handles complex sensor data, while the RNN focuses on immediate patterns, making the model computationally lighter and faster than CNN-LSTM. This combination can accurately predict errors that occur in motor drives of the robots, ensuring safety for surrounding people and preventing the robot from breaking down. The remainder of this paper is structured as follows: Section 2 presents the related work concerning various models of fault detection in the motor drive control systems. Section 3 presents the proposed methodology, which will cover current existing work of CNNs, Long Short-Term Memory networks (LSTMs), the combination of Convolutional Neural Networks and Long Short-Term Memory networks (CNN-LSTM), and the proposed model structure featuring a combination of Convolutional Neural Networks and Recurrent Neural Networks (CNN-RNN) [16,17,18]. The proposed solution flowchart and algorithm are also included in Section 3. Section 4 presents the simulation settings, parameters, and the existing work experimentation of error detection on motor drives of industrial robots. It also explains the experimentation of the proposed algorithm, the simulation setting, and its result. Finally, a conclusion of our work contributions and the significant tasks for future research are presented in Section 5.

## 2. Related Work

By integrating a variety of deep learning models, robotics engineers can employ numerous techniques to improve the precision and resilience of fault diagnosis in the context of motor drive control. In [19], the author proposed a novel approach to guarantee secure interaction between humans and robots in industrial settings. This entails the assessment of potential risks, the establishment of safety standards, and the implementation of real-time monitoring systems with the objective of preventing accidents. The key components of this approach include the setting of limits on robot movements, the utilization of sensors and machine learning to detect potential risks, and the implementation of adaptive safety mechanisms to ensure the safe interaction between humans and robots. The objective is to enhance productivity while simultaneously minimizing safety risks in collaborative manufacturing tasks. As stated in [20], the authors proposed an approach that utilized two camera systems with data fusion to merge information coming from the two devices and utilized Kalman filters to solve the obstruction problem and to compute human velocities and accelerations. This work could provide safety barrier functions and multi-camera tracking to ensure safe human–robot interaction in shared environments, using real-time tracking to adjust robot behavior and prevent collisions. Monitoring algorithms for industrial robots based on artificial intelligence and signal processing techniques was presented in [21,22]. Signal processing is essential in condition monitoring systems, as it helps extract key features from signals like vibration data to detect machine faults. Next is a novel method combining CNN and Extreme Learning Machine (ELM) for fast and accurate automated fault diagnosis in rotating machinery. The method processes raw vibration data using Continuous Wavelet Transform (CWT) to create scalograms, which are then input into a CNN for automatic feature extraction. In the second stage, ELM is used to enhance classification performance and learning speed. The method is validated on two datasets, gearbox and motor data, with various fault types and compared to other common methods, showing superior performance. In [23], the authors integrated IoT technology with vibration monitoring to diagnose faults in rotating machinery. Vibration data are collected by IoT sensors, processed to extract key features, and analyzed using machine learning algorithms for fault detection. The system enables real-time monitoring, sending alerts for maintenance, and storing data in the cloud for remote access and long-term analysis. Deep learning enhances fault detection and diagnosis by automatically analyzing complex sensor data to detect anomalies, classify faults, and monitor health in real time. By learning patterns in operational data, deep learning models can identify early warning signs of failures, enabling predictive maintenance and reducing downtime for more efficient and reliable operation [24,25,26,27].

As indicated in [28,29,30], intelligent fault diagnosis schemes based on LSTM networks had been proposed for use in rotating machinery and rolling bearings. The data-driven methods utilize measurement signals from multiple sensors, capturing both spatial and temporal patterns essential for fault diagnosis. By leveraging LSTM’s ability to process sequential data, these schemes effectively analyze the complex temporal dependencies within the data, providing accurate and reliable fault detection and diagnosis for machinery systems [28]. The authors proposed an early fault detection framework for rolling bearings using a stacked denoising auto-encoder (SDAE) and LSTM model. By analyzing vibration signals, the method detects initial faults based on reconstruction errors between actual and predicted data. Tested on real and benchmark datasets, it reliably identifies early anomalies and tracks degradation, offering strong support for maintenance planning [29]. Based on [30], the authors introduced an efficient method for early gear fault detection in rotating machinery using a hybrid feature set, combining Gamma Tone Cepstral Coefficient (GTCC) and Mel-Frequency Cepstral Coefficient (MFCC) from vibration signals. An LSTM classifier processes the temporal data, and tenfold cross-validation on two datasets demonstrates high accuracy. The results show that this feature fusion outperforms state-of-the-art methods, including those using pre-trained models, in detecting gear faults.

Next, CNNs are highly effective for fault detection and diagnosis, especially in complex machinery. They excel in automatically extracting and identifying features from raw data, such as vibration or image signals, which makes them suitable for identifying subtle fault patterns without extensive manual feature engineering [31,32,33]. A transfer learning method for rolling bearing fault diagnosis using simulated data and bearing fault diagnosis method that combines data enhancement and CNN to address issues in small sample scenarios were proposed in [34,35]. It decomposes vibration signals using Variational Mode Decomposition (VMD), selects relevant modes, and extracts features via autocorrelation. A four-layer CNN predicts fault types with high accuracy (99%) on the XJTU-SY dataset, requiring fewer training samples than existing methods. In [36], the authors presented a transfer learning method for rolling bearing fault diagnosis using simulated data, improving accuracy with limited real data. It also explores federated CNN with information fusion for fault diagnosis. Deep CNN outperforms traditional CNN in fault detection and diagnosis by learning more complex, hierarchical features through their deeper architecture. This enables better feature extraction, improved generalization across various fault types, and enhanced accuracy, particularly for subtle or early-stage faults in noisy data [37,38,39].

A recently researched model for fault detection and diagnosis is CNN-LSTM fault detection, which combines the feature extraction capabilities of CNN with the sequence learning strengths of LSTM networks. This hybrid model has shown superior performance compared to other existing models, as proposed in [40,41], due to its ability to capture both spatial and temporal patterns in the data, making it highly effective for detecting and diagnosing faults in complex systems. In [42], the author proposed a robust amplitude control set model predictive current control (RACSMPCC) method, which effectively enhances prediction accuracy and current control precision in PMSMs. This method reduces cost function values and compensates for disturbances through a nonlinear extended state observer (NESO). Furthermore, a parameter configuration method is introduced for NESO, which improves disturbance rejection. Hardware experiments validate the effectiveness of RACSMPCC in both reducing cost functions and improving current control accuracy. Next, the author introduced a novel cascaded adaptive deadbeat (CADB) control method for PMSM drives, which is designed to enhance dynamic performance and robustness. The method combines an adaptive deadbeat current controller, and a speed controller based on a simplified first-order current loop dynamic model. By identifying motor parameters through a gradient method, the necessity for additional observers is eliminated. The proposed controller’s stability is validated using the Lyapunov theorem, and experimental results demonstrate its excellent performance in steady and transient states as well as its robustness to disturbances [43]. According to [44], the author introduced a novel data-driven approach for predicting the remaining useful life (RUL) of tools employed in metal-forming processes. The combination of bidirectional LSTM (BLSTM) and CNN enables high accuracy in wear state classification and RUL estimation. The method outperforms other state-of-the-art techniques, demonstrating a low error rate (below 5%) and a root mean square error (RMSE) of approximately 1.5, which substantiates its robustness and precision in real-world applications. However, to obtain a more optimized model than CNN-LSTM, we have developed a new CNN-RNN model to achieve greater effectiveness, demonstrating stronger fault detection capabilities for industrial robot control systems compared to the existing CNN-LSTM-based method. The following section will explain how our model is created and how it outperforms other existing models, showcasing superior accuracy and precision in diagnosing faults in the DC motor drive of industrial robots.

## 3. Proposed Methodology

A proposes a hybrid fault diagnosis approach combining Convolutional Neural Networks and Recurrent Neural Networks (CNN-RNN) can solve challenges in detecting faults in complex systems for motor drives of industrial robots. CNN is used to extract spatial features from raw data, while RNN captures temporal dependencies in time-series data, allowing the model to analyze both the spatial and temporal aspects of system behavior. This hybrid model improves the accuracy and efficiency of fault detection, making it adaptable to a wide range of systems like machinery, robotics, and industrial processes. The method enhances fault diagnosis by considering both immediate patterns and changes over time, offering an efficient solution for predictive maintenance in complex systems.

### 3.1. System Architecture

**Figure 1:**
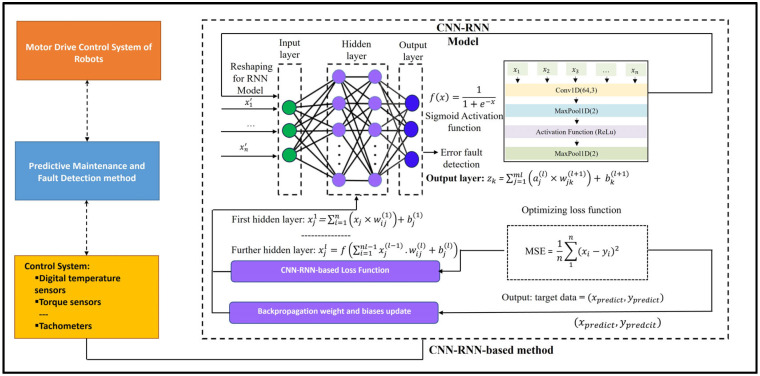
System architecture of the proposed model.

The Predictive Maintenance Dataset is used to model the motor drive control systems of industrial robots in our proposed work. The Predictive Maintenance Dataset is a synthetic dataset that mirrors real-world predictive maintenance data for industrial robots, and it is available for researchers. It is a data-driven approach that uses modeling to assess equipment condition and determine optimal maintenance timing. It helps prevent downtime, which can provide benefits in industries like manufacturing, transportation, energy, and healthcare, where equipment reliability is crucial for smooth operations. Figure 1 shows the system architecture of the proposed CNN-RNN model, which begins with sensors acting as the control systems to collect crucial data from the motor drive control systems of industrial robots. The important features to be collected include air temperature, process temperature, rotational speed, torque, and tool wear. These features are essential for monitoring the health of the robot’s motor drive system, enabling predictive maintenance and early fault detection.

The process of error detection in DC motor drives of industrial robots necessitates the analysis of sequential signals, including current, voltage, speed, torque, and vibration. These signals exhibit both spatial patterns and temporal dependencies, which must be considered for accurate detection. Both the CNN-RNN and CNN-LSTM architectures can leverage spatial and temporal analysis. However, the CNN-RNN can prove to be more effective than the CNN-LSTM due to its more straightforward design and suitability for the characteristics of motor drive error signals. The convolutional layers in both architectures are particularly adept at extracting spatial features, such as harmonics and anomalies, while the temporal models of LSTM process these features over time. RNN is effective for capturing moderate temporal dependencies, which is often sufficient for motor drive errors that manifest in short bursts or over moderate time spans. In contrast to LSTM, which is designed for complex long-term dependencies and involves computationally expensive gating mechanisms, RNN offers faster convergence, reduced complexity, and lower latency, rendering them optimal for real-time monitoring in resource-constrained industrial setups. Additionally, CNN-RNN can generalize more effectively by focusing on prominent sequential changes rather than overemphasizing long-term dependencies, which may not be critical for this specific task. Moreover, the more straightforward structure of RNN minimizes the probability of overfitting and improves explainability, which is a vital consideration for safety-critical systems such as industrial robots. The effectiveness of CNN-RNN can be experimented using motor drive datasets and evaluation metrics such as error-detected accuracy, recall, precision, and F1 -scores. When combined with preprocessing techniques such as wavelet transforms and attention mechanisms, CNN-RNN provides a robust and computationally efficient solution for real-time fault detection in industrial robots.

The following is the process of how the CNN-RNN model works in the entire predictive maintenance, fault detection, and diagnosis process for DC motor drive control for industrial robots:

Data Collection: The process begins with data collection from sensors integrated into the motor drive control systems of industrial robots. These sensors capture critical features like air temperature, process temperature, rotational speed, torque, and tool wear. These data points are collected continuously during the robot’s operation.

Data Preprocessing: The raw sensor data is processed to prepare it for machine learning models. This includes normalizing or scaling features, reducing noise to improve accuracy, and using data augmentation to handle class imbalances or limited data by creating new or resampled data.

Feature Extraction and Temporal Analysis using CNN-RNN: The preprocessed data are fed into CNN, which acts as a feature extraction mechanism. CNN is good at detecting spatial patterns, so they will identify critical features and correlations within the data (e.g., patterns in torque, temperature, or rotational speed). CNN layers help reduce the complexity of the data by extracting relevant spatial features, which can be used to detect anomalies and faults in the system’s operation. After feature extraction, the output from the CNN is passed to the RNN. The RNN is designed to handle sequential or time-series data, which is crucial for fault detection in systems like industrial robots, where faults may develop over time. The RNN captures the temporal dependencies between the features, allowing the model to recognize patterns or trends in the data that indicate potential faults.

Fault Diagnosis and Prediction: The CNN-RNN processes time-series sensor data to detect patterns and anomalies, such as sudden changes in torque or temperature, that indicate potential faults. It is trained to predict failures by identifying irregularities in the data.

Output of Fault Detection: After training, the integrated CNN-RNN model can make real-time predictions. When new data from the robot’s sensor system are input, the model classifies the system’s state into one of six classes: normal operation, power failure, tool wear failure, overstrain failure, heat dissipation failure, or random failure.

Decision Support for Predictive Maintenance: Finally, the model’s output helps in determining the optimal time for maintenance activities. If a fault is predicted, maintenance can be scheduled proactively, minimizing downtime and avoiding failures. The model’s predictions can trigger alerts to the maintenance team, who can investigate the issue before it leads to system failure.

### 3.2. Dataset Description

The dataset contains 10,000 data points, each representing a task used by motor drive control systems in industrial robots. Each data point is stored as a row with 14 features in columns that capture various aspects of the motor drive’s operation. These features include sensor readings and operational parameters, providing insights into the system’s performance and potential failure states. Air temperature is measured as Kelvin (K), which has the values corresponding to air temperatures of approximately 25 °C (298.1 K ≈ 25 °C and 298.2 K ≈ 25.05 °C). The air temperature is an important factor in motor performance, as it can influence the motor’s operating conditions and might affect the detection of faults, overheating, or thermal stress in the control systems. Process temperature is critical for detecting overheating, wear, or other issues directly affecting the robot’s motor and components. It typically refers to the temperature of a specific part of the system, such as the motor windings, bearings, or other critical components involved in the operation of the robot’s motor drive control systems. This temperature would be higher than the air temperature. The process temperature, with values of 308.6 K and 308.7 K, is approximately 35.5 °C and 35.6 °C, respectively.

Next, rotational speed refers to the speed at which the motor or a specific component (such as the robot’s drive system) is rotating, which is measured in revolutions per minute (rpm). Rotational speed is important for the proposed model because a sudden drop in speed could indicate a mechanical issue, a motor problem, or a control issue. Torque represents the torque exerted by the motor or the drive system during operation, which is measured in Newton meters (Nm). It is the rotational force that the motor applies to a mechanical load, such as the robot’s arm or any moving part. Sudden spikes in torque might indicate that the motor is compensating for mechanical resistance. Unexpected drops in torque may signal power issues, a reduction in load, or even a failure in the drive system. Also, abnormally high torque could also indicate a motor overload, which can lead to overheating or damage to components. Finally, tool wear refers to the amount of usage time in minutes that the tool (like a drill bit, cutting edge, or other mechanical component) has spent for the completion of that task, which is measured in minutes. Over time, tools in industrial robots show degradation due to friction, heat, and mechanical stress. By analyzing these features, one can gain a deeper understanding of the factors influencing the motor drive’s behavior and the likelihood of system failure under different conditions.

**Figure 2:**
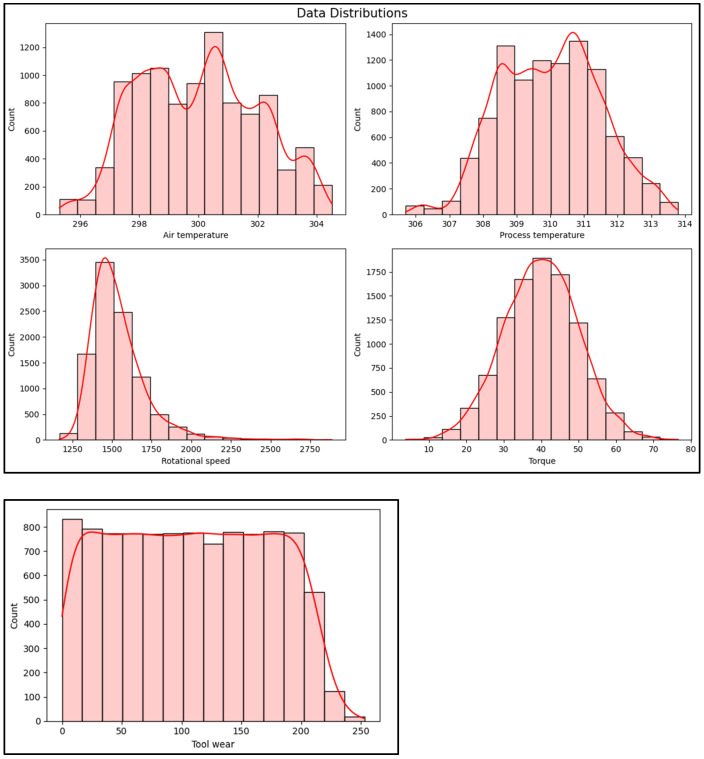
Data destitution.

Figure 2 shows the data destitution, which has features like air temperature, process temperature, rotational speed, torque, and tool wear that exhibit unique data distributions that are essential for effective fault prediction. Air temperature often follows a normal distribution, reflecting typical ambient variations, while process temperature generally shows a positive skew due to the heating effects within machinery during operation. Rotational speed commonly displays a bimodal or multimodal distribution as machines operate at varying speeds based on task demands. Torque is also positively skewed, influenced by fluctuating resistance and power demands, whereas tool wear shows a steady right-skewed increase, representing gradual tool degradation over time.

**Figure 3:**
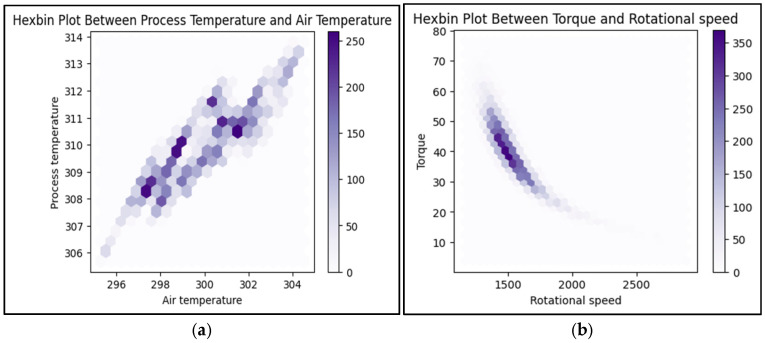
The hexbin plot between process temperature and air temperature (**a**); the hexbin plot between torque and rotational speed (**b**).

Figure 3 shows the hexbin plot between process temperature and air temperature, showing their relationship, often indicating a slight positive correlation where the process temperature varies more widely than air temperature, helping to spot potential overheating. For torque and rotational speed, the hexbin plot highlights typical operating ranges, showing that torque often rises with speed up to a point. Outliers in these plots, like high torque at low speeds, may indicate mechanical issues, aiding in fault detection and understanding machine performance.

### 3.3. Existing Research and Proposed Fault Detection Algorithms

In fault detection research, various models have been applied to improve predictive maintenance. Methods include Random Forest for robust detection, Adaptive Boosting and CatBoost for enhanced accuracy with categorical data, and Gradient Boosting for complex pattern recognition. Deep learning models like CNN can capture spatial patterns, while LSTM is effective for temporal data. Hybrid approaches, such as CNN-LSTM models, integrate spatial and sequential insights, enhancing the accuracy of fault predictions and contributing to more reliable maintenance strategies. The following section will explain each model along with our newly proposed CNN-RNN method.

#### 3.3.1. Traditional and Existing Research Models

For fault detection, a range of machine learning models are frequently used, each with its own distinctive set of capabilities. The Random Forest method is an ensemble approach that constructs multiple decision trees, combining their outputs to enhance accuracy and robustness. This makes it an effective tool for identifying patterns in both structured and unstructured data. Adaptive Boosting (AdaBoost) is a method that enhances the performance of weak learners by iteratively focusing on instances that have been misclassified. This method makes it a useful technique for detecting subtle faults in complex systems. CatBoost, a gradient boosting algorithm designed for categorical data, reduces the time and effort required for preprocessing and is particularly effective when features like equipment type are categorical. Gradient Boosting combines weak models to create a strong predictive model, excelling at handling complex relationships and nonlinear patterns in data. Finally, XGBoost (version 1.7.0) is an effective machine learning algorithm for fault detection in motor drive control systems of industrial robots. It provides high accuracy, computational efficiency, and the ability to handle complex, noisy data. The process involves collecting and preprocessing data from sensors (e.g., current, voltage, temperature), creating features, and training the XGBoost model using labeled normal and faulty data. Once trained, the model can classify real-time data to detect potential faults, allowing for timely interventions. XGBoost’s feature importance insights help identify critical parameters contributing to faults, enhancing system reliability and reducing operational downtime. Collectively, these models are valuable tools for fault detection, which is capable of identifying patterns and improving prediction accuracy through iterative learning and ensemble techniques.

#### 3.3.2. Long Short-Term Memory (LSTM) Networks

The core concept of LSTM is the incorporation of memory cells that can store and retrieve information over extended periods. The memory cells are specifically designed to store information over prolonged periods, allowing the network to learn and retain patterns within the input sequence. The LSTM architecture is composed of three principal components: the input gate, the forget gate, and the output gate. The gates regulate the flow of information within the network, determining which parts of the input should be retained, discarded, or output. As a result, LSTM networks are able to effectively capture long-term dependencies in input sequences, leading to more accurate predictions or classifications, particularly in time-series data [45]. The LSTM network is a sequence-to-sequence model, which is well suited for tasks such as condition monitoring, language modeling, speech recognition, and sentiment analysis. In LSTM, the context of previous inputs is crucial for understanding the current input due to its ability to transmit information across sequences. The Long Short-Term Memory network addresses the issue of long-term dependence by incorporating memory cells, which enable the network to selectively retain or discard previous input information through the regulation of information flow. The LSTM cell comprises three gates: the forget gate, the input gate, and the output gate, which are represented by the following symbols: $f_{g},i_{g},o_{g}$, respectively.

(1) Forget Gate: The forget gate ($f_{g}$) determines the degree to which information from the previous cell state is retained or discarded. This can be expressed as follows:

(1) $$f_{g}=\sigma \left(\;W_{f}\times \left[\;h_{g-1},\;x_{g}\;\right]+b_{f}\right)$$

(2) Input Gate: The input gate determines the importance of the incoming data, thereby influencing the extent to which this information is retained. This can be expressed as follows:

(2) $$\left\{\begin{array}{c} i_{g}=\sigma \left(\;W_{g}\times \left[\;h_{g-1},\;x_{g}\;\right]+b_{i}\right)\;\;\;\;\;\;\;\;\;\;\;\;\;\;\;\;\;\;\;\;\;\;\;\;\;\;\;\;\;\;\;\;\;\;\;\;\;\;\;\;\;\;\;\;\;\;\\ C_{g}=f_{g}\times C_{g-1}+tanh\left(\;W_{C}\times \left[\;h_{g-1},\;x_{g}\;\right]+b_{c}\right)\times i_{g} \end{array}\right.$$

(3) Output Gate: The output gate determines which information is emitted from the cell state, as expressed by the following formula:

(3) $$\left\{\begin{array}{c} o_{g}=\sigma \left(\;W_{o}\times \left[\;h_{g-1},\;x_{g}\;\right]+b_{o}\right)\\ h_{g}=o_{g}\times \tanh\left(C_{g}\right)\;\;\;\;\;\;\;\;\;\;\;\;\;\;\;\;\;\;\;\;\;\; \end{array}\right.$$

Here, $C_{g}$ and $C_{g-1}$ represent the current memory cell state at time step t and the previous memory cell state at time step t−1, respectively; $h_{g}$ and $h_{g-1}$ represent the current output state and the previous output state at time steps t and t−1, respectively; $W_{f},\;W_{i},W_{c},\;and\;W_{o}$ represent the weights associated with each gate. Next, $b_{f},b_{i},\;b_{c},and\;b_{o}$ are the corresponding biases of each gate. In this context, σ represents the sigmoid function, while tanh denotes the hyperbolic tangent activation function [3,45].

#### 3.3.3. Convolution Neural Networks

CNN is effective in image recognition, computer vision, and fault diagnosis due to its efficient architecture, which combines local perceptual fields, weight sharing, and downsampling. These strategies reduce model complexity and enable CNN to detect intricate spatial patterns, making them ideal for high-dimensional tasks. A typical CNN consists of convolutional layers for feature detection, pooling layers for dimensionality reduction, and fully connected layers for final classification [46,47,48].

CNN uses a series of filters, or kernels, to process input data and identify patterns. Each filter performs a dot product between its weights and localized regions of the input, resulting in a feature map that highlights specific features within the data [49,50]. This feature map summarizes where particular patterns, like edges or textures, are detected in the input. The convolution operation, which creates these feature maps, can be expressed mathematically as follows:

(4) $$Y\left(j,k\right)=\left(X\times W\right)\left(j,k\right)\;=\sum_{m=1}^{M}\sum_{n=1}^{N}X\;\left(j+m,k+n\right)\times W(m,n)$$

In this equation, $Y\left(j,k\right)$ represents the output at position $\left(j,k\right)$ in the feature map; X is the input data; W is the filter; and m and n are the dimensions of the filter.

The pooling layers reduce the spatial dimensions of the feature maps by performing downsampling operations, such as max or average pooling, with the goal of mitigating overfitting. The adopted max pooling formula can be described as follows:

(5) $$Y\left(j,k\right)=\underset{m=1}{\max}\underset{n=1}{\max}X(\left(j-1\right)\times s+m\left(k-1\right)\times s+n$$

Here, $m\;and\;n\in \;k^{'}$, X represents the input feature map, Y represents the output feature map after pooling, k is the size of the pooling kernel, and s is the pooling stride. In the case of max pooling, $Y\left(j,k\right)$ represents the pooled feature value located at row j and column k. This value is equal to the maximum value in the $k^{'}\times k^{'}$ input feature map, starting from the row coordinate (j−1) and column coordinate (k−1).

After the convolutional and pooling layers, the fully connected layer consolidates the extracted feature information, enabling the model to integrate and interpret features across different classes for accurate classification. In this layer, each neuron is connected to every neuron in the previous layer, allowing it to process the entire set of features and make final predictions. This dense connectivity gives the network a more global understanding of the data, ultimately enabling precise class distinctions based on the learned patterns.

(6) $$\left\{\begin{array}{c} \;\;Y=W\times X+B\;\;\;\;\;\;\;\;\;\;\;\;\;\;\;\;\;\\ \;\;y_{j}=\frac{e^{z_{j}}}{\sum_{k=1}^{k^{'}}e^{z_{j}}}\;\;\;\;\;\;\;\;\;\;\;\;\;\;\;\;\;\;\;\;\; \end{array}\right.$$

In this context, Y represents the output vector, W denotes the weighting matrix, X signifies the input vector, B is the bias vector, $z_{j}$ represents the unnormalized score for the i-th output class; $y_{j}$ represents the probability of the i-th output class; and $k^{'}$ is the total number of output classes.

#### 3.3.4. CNN-LSTM Fault Diagnosis Model

The LSTM network effectively addresses the challenges of long-term dependency and vanishing gradients in RNN, making it ideal for time-series data. In contrast, CNN is efficient at local feature extraction and has faster convergence but is less effective with sequential data, which limits their performance for time-sensitive tasks. Given that the fault data in this experiment include temporal information and interconnected fault types where one fault could trigger or influence another, the proposed CNN-LSTM model is constructed for more accurate fault diagnosis in heavy-duty industrial robots [40]. This model first uses CNN layers to extract local features, which is followed by LSTM layers to capture temporal features, thereby balancing the extraction of time-dependent information with accelerated training. This approach is then followed by LSTM to prioritize the sequential processing of data. The CNN-LSTM model comprises three layers: the LSTM layer, the CNN layer, and the output layer, as illustrated in Algorithm 1.

The process of building and evaluating a CNN combined with an LSTM model for binary classification of machine failure includes the following steps. The data are first preprocessed by splitting it into input features (X) and the target variable (Y), which is followed by scaling the features using StandardScaler to ensure uniformity. Since LSTM requires a 3D input format, the data are reshaped accordingly. The model consists of a 1D convolutional layer with ReLU activation to extract features, which is followed by a max-pooling layer to reduce dimensionality. Regularization is utilized with a dropout layer to prevent overfitting. The LSTM layer is then added to capture long-range dependencies in the data, which is followed by a fully connected dense layer and an output layer with a sigmoid activation function for binary classification. The model is compiled with the Adam optimizer and binary cross-entropy loss, and then it is trained with early stopping to prevent overfitting. After training, the model’s performance is evaluated using metrics such as mean absolute error (MAE), prediction accuracy, recall, F1-score, and precision. The confusion matrix is also computed to assess the model’s classification results.
**Algorithm 1 CNN-LSTM-based**
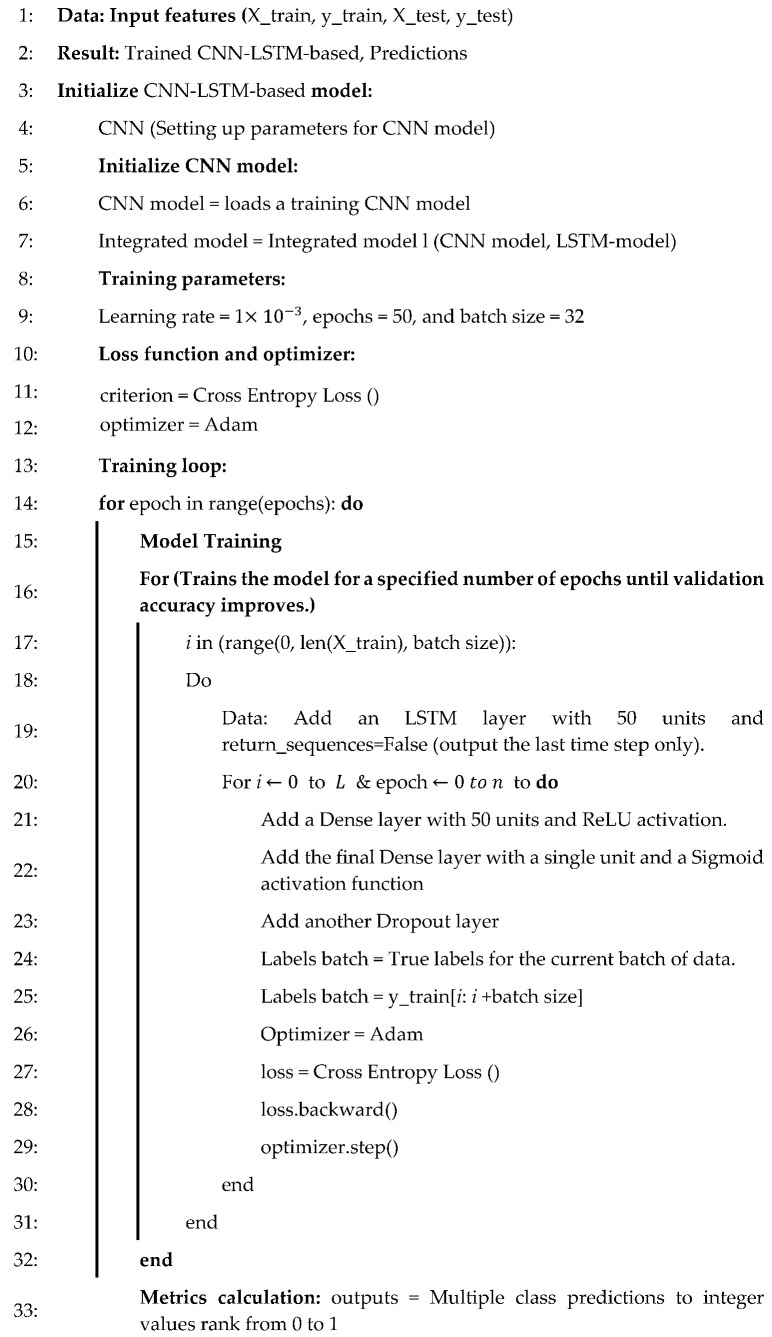


The hybrid CNN-LSTM model for predictive maintenance and fault detection in motor drive control systems combines convolutional layers to extract features with an LSTM layer to capture sequential dependencies in the data. The train_test_split function from sklearn.model_selection divides a dataset into training and testing sets. In this example, ($X_{train}\;$(input data) and (y_train (target labels) are split with 80% of the data allocated to training (X_train, y_train) and 20% to testing (X_test, y_test). The test_size with a value of 0.2 specifies the size of the test set, while random_state with a value of 42 ensures consistent shuffling for reproducibility. This setup allows the model to be trained on one subset and evaluated on prediction data to assess its performance. Key hyperparameters include 64 filters with a kernel size of 3 for the Conv1D layer, 50 units in the LSTM layer, and 50 units in the dense layer with ReLU activation. The model uses Adam optimizer and binary cross-entropy loss with accuracy as the evaluation metric. Regularization is applied through dropout layers (with a rate of 0.2) and early stopping to prevent overfitting. The model is trained for up to 50 epochs with early stopping and a batch size of 32. By integrating CNN and LSTM layers, the CNN-LSTM model first processes raw signals with the CNN to detect relevant feature patterns; then, it passes these patterns to the LSTM, which models the temporal behavior of the extracted features over time. This approach enables the model to not only recognize spatial patterns indicative of fault conditions but also track their evolution, allowing for accurate predictions of future states and timely fault detection. This combination is particularly advantageous in industrial robot maintenance, where detecting early signs of failure and understanding long-term trends are essential for proactive maintenance and reducing downtime.

#### 3.3.5. Recurrent Neural Networks (RNN)

LSTM has more complex architectures with additional components like input, forget, and output gates. This increases computational requirements and can slow down training and inference, making them overkill for tasks that do not require handling long-term dependencies. RNN can be highly effective when dealing with tasks that only require learning short-term dependencies in the data. It performs well in scenarios where relationships between data points are close in sequence and where long-term context is not necessary. In cases where the data exhibit simpler patterns or periodic behaviors that do not require complex temporal reasoning, RNN can be sufficient and often perform just as well as LSTM. It can excel in structured or predictable data where simpler recurrent architectures are enough. Because of their simpler structure, RNN can converge faster during training for specific tasks where capturing basic sequential information is enough. This can make RNN a better choice for quick prototyping or when experimenting with initial models to obtain a baseline performance. The lower parameter count in RNNs can make them less prone to overfitting when working with smaller datasets. LSTM, with its added complexity and parameters, can overfit if the dataset does not have enough information to justify the model’s complexity. In summary, RNN can outperform LSTM in scenarios where the task only requires handling short-term dependencies, when computational and memory efficiency are priorities, or when the dataset is small and simple. Their simpler design allows for faster processing, making them ideal for straightforward, real-time applications where complexity might not add value. While LSTM is more powerful for complex temporal patterns, RNN provides a more efficient and pragmatic solution for simpler sequential data problems.

#### 3.3.6. Proposed CNN-RNN Method

Integrating CNN with RNN for predictive maintenance and error detection on the motor drive control of industrial robots provides a robust solution due to the combined strengths of CNN’s feature extraction capabilities and RNN’s temporal analysis. The following are detailed benefits of how this integration enhances robustness, especially considering the differences between CNN-RNN and CNN-LSTM. CNN is exceptional at extracting spatial patterns from raw data, such as sensor readings, vibrations, or images related to motor drive behavior. In predictive maintenance, CNN layers can identify key features like frequency components or anomalies in sensor signals that might indicate early signs of wear, imbalance, or other mechanical faults. When CNN is integrated into an RNN, the CNN acts as a powerful preprocessing step, transforming raw data into high-level features that are more meaningful for fault detection. This enables the RNN to focus on temporal relationships without being overwhelmed by the complexity of raw input data.

RNN Efficiency: RNN is particularly efficient in analyzing short-term temporal dependencies, where relationships between data points are close in sequence. In motor drive control systems, this means RNN can effectively detect patterns that occur over short time frames, such as sudden changes in motor current, temperature fluctuations, or vibration spikes. Short-Term Focus: For predictive maintenance tasks that do not require long-term dependencies, such as monitoring real-time operational status or detecting immediate faults, RNN is sufficient and more computationally efficient than LSTM. This reduces the risk of overfitting, making the model reliable even with smaller datasets. This complexity can be unnecessary for real-time industrial monitoring, where faults often occur through immediate changes in sensor data. RNN Advantage: Integrating CNN with a simpler RNN architecture allows for faster training and inference, which is crucial in real-time predictive maintenance applications. A CNN-RNN model is computationally lighter than a CNN-LSTM, making it suitable for environments with limited processing power, like edge computing devices attached to industrial robots. Motor drives in industrial robots often exhibit structured behaviors, such as periodic vibrations, regular current cycles, or consistent temperature patterns. RNN is well suited for this type of predictable, structured data, where simpler recurrent architectures can perform effectively. LSTM might introduce unnecessary complexity in these cases, potentially leading to slower processing times and increased memory usage. The RNN’s straightforward structure allows for more efficient handling of these predictable patterns without sacrificing accuracy. Because RNN converges faster during training due to their simpler architecture, integrating them with CNN can speed up the development cycle. This makes CNN-RNN models an excellent choice for quick prototyping or iterative experimentation, allowing engineers to refine models for specific motor drive fault scenarios quickly.

In summary, by integrating CNN with RNN, the robustness of predictive maintenance and fault detection models for industrial robot motor drives is significantly enhanced. CNN efficiently handles feature extraction from complex sensor data, while the RNN focuses on analyzing short-term temporal dependencies without the computational burden of LSTM. This combination offers a practical balance between accuracy and efficiency, making it ideal for real-time applications where speed and simplicity are crucial while still maintaining the ability to detect faults accurately in structured, predictable data. The CNN-RNN model is a pragmatic and robust solution for industrial environments, ensuring reliable fault detection and proactive maintenance with minimal computational demands. Algorithm 2 illustrates the integration of CNN with RNN for predictive maintenance and fault detection in DC motor drives of industrial robots. It provides a robust solution by combining CNN’s feature extraction capabilities with RNN’s temporal analysis strengths.

Algorithm 2 illustrates the proposed CNN-RNN observers. The architectural details are fully elaborated with the specific hyperparameters along with an analysis of the strengths and potential for outperformance of the current existing CNN-LSTM model for error detection on motor drive control of industrial robots. The input data, which have been scaled using the StandardScaler function, are reshaped into a three-dimensional array with a shape of (samples, features, 1) for the Conv1D layer. In this context, the term “samples” denotes the number of data points, whereas “features” refers to the number of features present in each data point. This reshaping ensures that the data are compatible with the Conv1D layer, which expects a 3D tensor where the last dimension corresponds to the number of channels (in this case, 1 channel due to reshaping). The convolutional layer (CNN) comprises 64 filters, enabling the learning of 64 distinct feature maps from the input data. The kernel size is set to 3, indicating that the convolutional filter will scan over three consecutive time steps (or features) at a time. The activation function employed is ReLU (Rectified Linear Unit), which introduces nonlinearity into the model and facilitates the learning of complex patterns. The input shape is determined dynamically in accordance with the number of features present in the input data with each feature represented by a single channel. The max pooling layer serves to reduce the dimensionality of the dataset by selecting the maximum value from each pool of size 2 along the feature dimension, which is to say the time step axis. The pool size of two signifies that the layer will undertake a downsampling of the data by a factor of two, thereby reducing the number of features for the subsequent layer. This facilitates a reduction in the computational load and the extraction of the most pertinent features from the convolutional output. The dropout layer randomly sets 20% of the units in the preceding layer to zero during the training phase, thereby forcing the model to rely on different features at each step. A dropout rate of 0.2 is beneficial in preventing the model from overfitting to the training data, thereby enhancing its capacity to generalize to prediction data. The RNN layer comprises 32 units, enabling the learning of 32 distinct features from the sequential data. RNN units are effective in capturing long-term dependencies in sequential data, rendering them well suited for time-series or sequence-based problems. The parameter returns sequences to false, ensuring that the RNN layer outputs solely the final hidden state (as opposed to the entire sequence of hidden states), which is appropriate for tasks such as binary classification where a single output per sequence is required. The dense layer (output layer) contains a single unit, as the task is binary classification, necessitating only one output. The sigmoid activation function is employed, which outputs a value between 0 and 1, representing the probability of the positive class. This configuration is optimal for binary classification tasks, wherein the model must determine whether the input data falls within one of two predefined classes (in this case, failure or no failure).
**Algorithm 2 CNN-RNN-based**
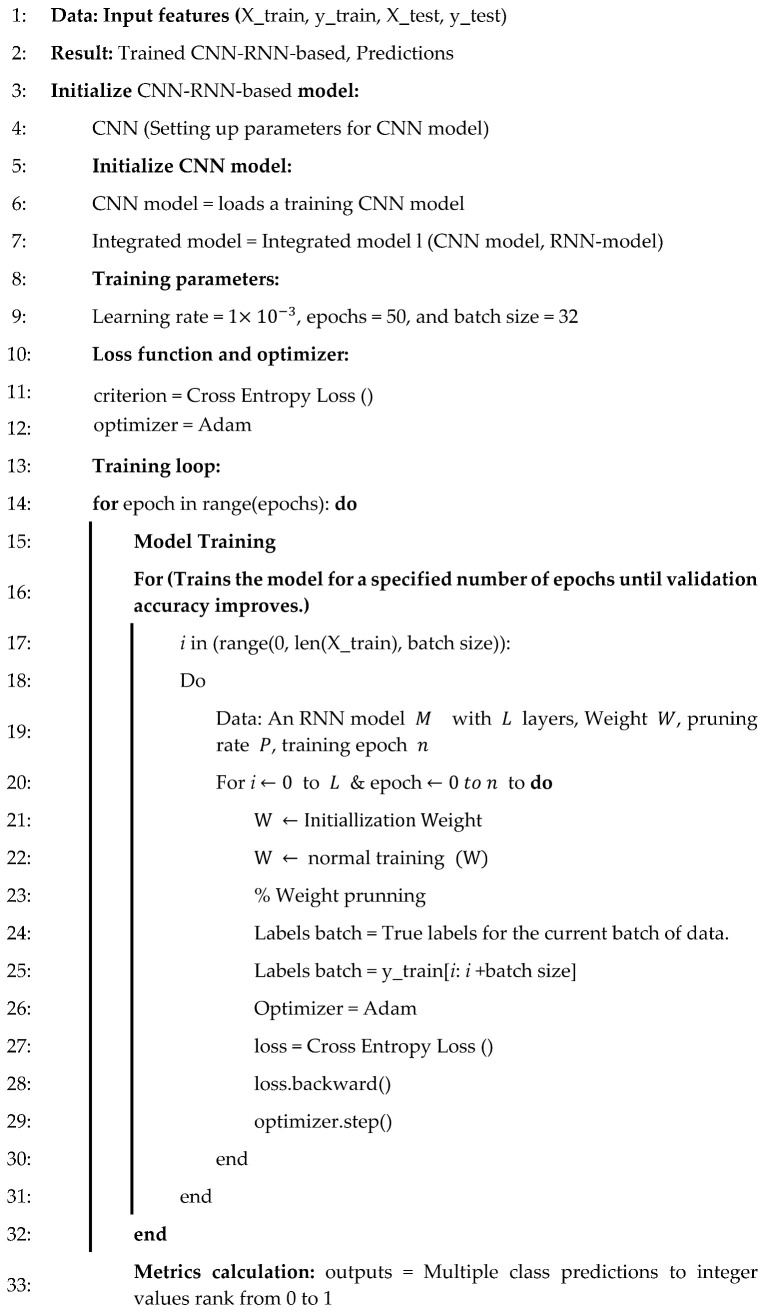


The training hyperparameters are as follows: The Adam optimizer is a type of optimization algorithm that is used to find the best parameters for a given problem. This is an adaptive optimizer that modifies the learning rate during the training phase, thereby enhancing efficiency for complex models. The loss function is as follows: Binary cross-entropy is an appropriate loss function for binary classification problems. The model tracks accuracy during training, thereby providing insights into both classifications. The model is trained for 50 epochs, which is the number of times the model will iterate over the entire dataset during training. The batch size is set to 32 samples per batch, which determines how many samples are processed before the model’s weights are updated.

## 4. Experiment Result and Analysis

To ensure the effective implementation of the CNN-RNN algorithm, it is essential to use the traditional CNN model with the connection to an RNN. The objective of this integration is to enhance the model’s capacity to identify and capture the effectiveness of fault detection for motor drive control systems of industrial robots. The dataset, which is made for its binary classification tasks during training, will serve as the primary benchmark for evaluating the algorithm’s performance. The objective is to enhance the model’s accuracy and robustness in analysis by merging CNN’s existing model with RNN’s sequential learning capabilities. The following section provides a detailed explanation and analysis of the evaluation metrics, the proposed method, and a comprehensive overview of existing work of error detection on motor drives of industrial robots. The objective of this section is to provide a comprehensive understanding of the methodologies employed, offering insights into the strengths and limitations of the proposed approach in comparison with previously established models.

### 4.1. Datasets and Evaluation Metrics

In this paper, the Predictive Maintenance Dataset [16] is selected as the experimental dataset, which is further described in detail in Section 3.2. The dataset is split into a training set comprising 80% of the data and a testing set containing the remaining 20%. It consists of 10,000 data points, each representing a specific task performed by motor drive control of industrial robots. Each data point is stored as a row with 14 features in columns that capture various critical aspects of the motor drive’s operation. These features include sensor readings, operational parameters, and other relevant factors that provide valuable insights into the system’s performance and potential failure states. The main goal of this study is to predict whether the motor of the robot is in a faulted state or operating normally, which plays a crucial role in ensuring the reliability and efficiency of industrial robots in real-world applications.

In classification problems, metrics such as accuracy, precision, recall, and F1-score are typically employed to assess the performance of classification models. These metrics provide important insights into how well the model is distinguishing between different classes. According to the confusion matrix, the experimental results can be classified into four categories: true positive (TP), false positive (FP), true negative (TN), and false negative (FN). Among these, TP denotes the number of instances with positive actual labels and positive model predictions; FP denotes the number of instances with negative actual labels but positive model predictions; FN denotes the number of instances with positive actual labels but negative model predictions; and TN denotes the number of instances with negative actual labels and negative model predictions. These four categories form the basis for evaluating the model’s performance. The accuracy, precision, recall, and F1-score are calculated based on these values, allowing for a comprehensive assessment of how well the model is performing, particularly in distinguishing between different classes or detecting specific patterns within the data. The following formulas describe how these metrics are derived:

(7) $$Accuracy=\frac{TP+TN}{TP+FP+TN+FN}*100\%$$

(8) $$Precision=\frac{TP}{TP+FP}*100\%$$

(9) $$Recal=\frac{TP}{TP+FN}*100\%$$

(10) $$F1score=\frac{2*Precision*Recal}{Precision+Recal}*100\%$$

The above formula indicates that accuracy is the ratio of correctly classified samples to the total number of samples. This reflects the overall performance of the classifier. However, if there is a category imbalance in the dataset, the accuracy rate may be distorted because the classifier may tend to predict the category with a higher number of samples. Accuracy is the percentage of samples that the classifier predicts to be positive that are actually positive cases. The recall rate is the proportion of positive examples that are correctly identified as such by the classifier. When the samples are unbalanced, the precision and recall rates can give a more accurate reflection of the classification performance of the model, although they may not fully capture the predictive ability of negative samples. The F1-score is the average of the precision and recall rates, which can combine the prediction accuracy and recognition ability of the classifier. However, the F1-score gives equal weight to precision and recall, which may result in overlooking certain metrics that should be given more weight. In summary, each of the four metrics, such as accuracy, precision, recall, and F1-score, has its own advantages and disadvantages. To comprehensively evaluate the classification performance of the model, the above four metrics are adopted as the evaluation metrics for this experiment.

### 4.2. Experiments and Parameter Settings

The model consists of the integration of CNN and RNN to predict the error detection on motor drives of industrial robots. The dataset is first split into training and test sets (80% and 20%, respectively) and scaled using the StandardScaler to standardize the features. The reshaped data are then processed by a 1D CNN layer with 64 filters and a kernel size of 3, which is followed by a max pooling layer with a pool size of 2. This convolutional part of the model extracts relevant spatial features from the input data, which is followed by a 20% dropout layer to reduce overfitting.

The output of the CNN is passed to an RNN layer with 32 units, which captures temporal dependencies in the data. The RNN layer’s output is then fed into a dense layer with a single neuron and a sigmoid activation function, enabling binary classification (fault vs. normal operation). The model is compiled using the Adam optimizer with a binary cross-entropy loss function, and performance is measured using accuracy, precision, recall, and F1-score.

The model is trained for 50 epochs with a batch size of 32, using the training data while monitoring performance on the test set for validation. After training, the model’s predictions are evaluated using classification metrics such as precision, recall, F1-score, confusion matrix, mean squared error, mean absolute error, and standard deviation of predictions. Lastly, training and validation accuracy/loss are plotted to visualize the model’s learning process and performance over epochs. The details of the important parameters are shown in Table 1.

### 4.3. Results and Discussion

#### 4.3.1. Proposed CNN-RNN-Base and Traditional Models

**Figure 4:**
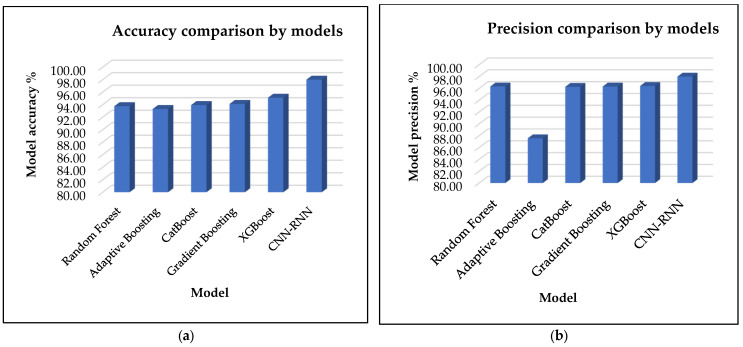
A comparison of CNN-RNN model’s accuracy with other methods (**a**); a comparison of CNN-RNN model’s precision with other methods (**b**).

Figure 4a presents the results of the proposed model, CNN-RNN, in comparison to traditional models, including Random Forest, Adaptive Boosting, CatBoost, Gradient Boosting, and XGBoost, in terms of accuracy.

The results demonstrate that the proposed model exhibits a higher level of accuracy than other models with a score of 98.00%. The XGBoost model demonstrates the highest accuracy, at 95.11%, but it stands behind the CNN-RNN. The Gradient Boosting, CatBoost, and Random Forest models achieve accuracies of 94.11%, 93.93%, and 93.75%, respectively. However, the Adaptive Boosting model exhibits a comparatively lower performance with an accuracy of only 93.30%.

Figure 4b presents the results of the proposed model, CNN-RNN, in comparison to traditional models, including Random Forest, Adaptive Boosting, CatBoost, Gradient Boosting, and XGBoost, in terms of model precision. The results demonstrate that the proposed model exhibits a higher level of precision than other models with a score of 98.00%. The XGBoost model demonstrates the highest accuracy at 95.11%, but it stands behind the CNN-RNN. The Gradient Boosting, CatBoost, and Random Forest models achieve precisions of 96.32%, 96.27%, and 96.33%, respectively. However, the Adaptive Boosting model exhibits comparatively lower performance than other models with a precision of only 93.30%.

**Figure 5:**
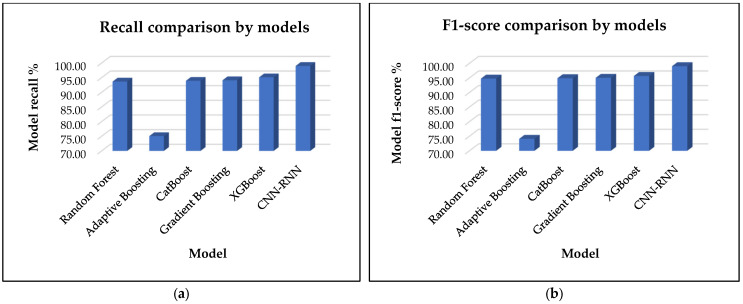
A comparison of CNN-RNN model’s recall with other methods (**a**); a comparison of CNN-RNN model’s F1-score with other methods (**b**).

Figure 5a illustrates the comparative performance of the proposed model, CNN-RNN, against several traditional machine learning models, including Random Forest, Adaptive Boosting, CatBoost, Gradient Boosting, and XGBoost, specifically focusing on recall metrics. The analysis indicates that the CNN-RNN model significantly outperforms the traditional models, achieving a recall of 99.00%. This high recall highlights the effectiveness of integrating convolutional and recurrent neural network layers for the classification task at hand. Among the traditional models, XGBoost achieves the best performance with an accuracy of 95.11%, showing it as a strong competitor though still falling short of the proposed model. Gradient Boosting follows closely with an accuracy of 94.12%, while CatBoost and Random Forest attain recalls of 93.93% and 93.68%, respectively. In contrast, the Adaptive Boosting model stands behind with the lowest recall of 75.05%. This suggests that while boosting techniques generally offer competitive performance, they may not capture the underlying progression as effectively as the hybrid deep learning architecture utilized in the CNN-RNN model.

Figure 5b depicts the comparative performance of the proposed model, CNN-RNN, in relation to several traditional machine learning models, including Random Forest, Adaptive Boosting, CatBoost, Gradient Boosting, and XGBoost with a particular emphasis on F1-score metrics. The results of the analysis demonstrate that the CNN-RNN model exhibits a markedly superior performance compared to the traditional models with an F1-score rate of 99.00%. This high recall value demonstrates the effectiveness of integrating Convolutional Neural Network and Recurrent Neural Network layers for the classification task at hand. Among the traditional models, XGBoost achieves the highest accuracy (95.65%), demonstrating its potential as a competitive alternative, although it still falls short of the proposed model. Gradient Boosting exhibits a similar level of accuracy with a score of 95.02%. CatBoost and Random Forest demonstrate comparable recall rates with values of 94.89% and 94.76%, respectively. In contrast, the Adaptive Boosting model exhibits the lowest recall at 74.10%. This indicates that while boosting techniques typically demonstrate competitive performance, they may not effectively capture the underlying progression as much as the hybrid deep learning architecture employed in the CNN-RNN model.

#### 4.3.2. Proposed CNN-RNN-Base and Other Current Existing Research

**Figure 6:**
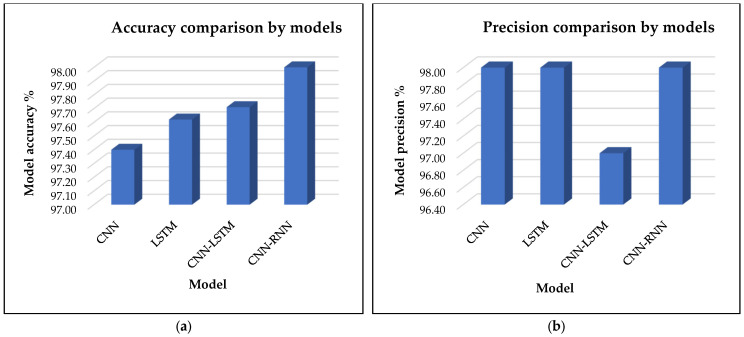
A comparison of CNN-RNN model’s accuracy with recent existing works (**a**); a comparison of CNN-RNN model’s precision with recent existing works (**b**).

Figure 6a presents a comparative analysis of the proposed CNN-RNN model against existing research models, including CNN, LSTM, and CNN-LSTM, focusing specifically on accuracy. The results clearly indicate that the proposed CNN-RNN model achieves superior performance with a remarkable accuracy of 98.00%. This highlights the effectiveness of combining convolutional and recurrent layers, enabling the model to capture both spatial and sequential features with greater precision. The CNN-LSTM model ranks as the second most accurate, achieving an accuracy of 97.71%, closely trailing the CNN-RNN but still demonstrating the benefits of integrating convolutional and RNN structures. Meanwhile, the LSTM model reaches an accuracy of 97.62%, demonstrating the strong capabilities of recurrent networks for capturing temporal dependencies. Finally, the standalone CNN model, while effective, shows a slightly lower accuracy of 97.40%, indicating that although convolutional layers are excellent at extracting spatial features, their performance improves when complemented with sequential processing components. Overall, the results highlight the advantage of the hybrid CNN-RNN architecture, which leverages the strengths of both CNN and RNN models, outperforming traditional deep learning approaches in accuracy.

Figure 6b presents a comparative analysis of the proposed CNN-RNN model against existing research models, including CNN, LSTM, and CNN-LSTM, with a particular focus on precision. The results clearly demonstrate that the CNN-RNN, LSTM, and CNN models exhibit superior performance, each achieving a precision score of 98.00%. This suggests that these models are highly effective at minimizing false positives, making them reliable for accurate classification in this specific context. In contrast, the CNN-LSTM model achieves a slightly lower precision with a score of 97.00%. The lower precision of the CNN-LSTM model compared to the other models can be attributed to its inherent complexity. While the CNN-LSTM model combines the strengths of both convolutional and recurrent layers, leveraging CNN’s spatial feature extraction and LSTM’s ability to capture temporal dependencies, this complexity can sometimes lead to overfitting. The CNN-LSTM model may pick up on subtle patterns in the training data that do not generalize well to prediction data, resulting in a higher number of false positives, which negatively impacts precision. Additionally, the interaction between the convolutional and sequential layers may introduce noise or redundant features that do not contribute meaningfully to classification, thus slightly reducing the model’s precision.

In comparison, the CNN-RNN model, despite also combining convolutional and recurrent components, appears to handle feature extraction and sequence learning in a more balanced manner. This balance allows it to maintain high precision, as it effectively distinguishes relevant patterns without overfitting. Similarly, the standalone CNN and LSTM models, though less complex, demonstrate robust precision due to their focused architectures that excel in their respective domains: CNN for spatial data and LSTM for temporal sequences, resulting in fewer false positives and higher precision scores.

**Figure 7:**
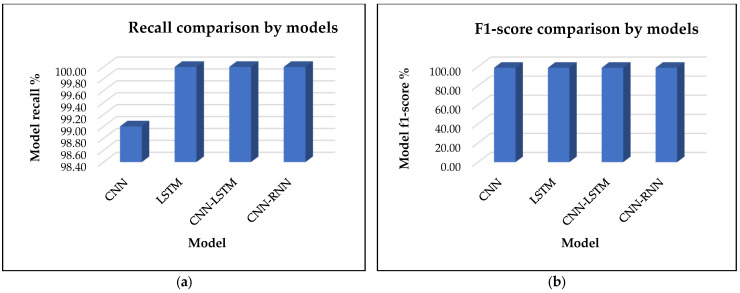
A comparison of CNN-RNN model’s recall with recent existing works (**a**); a comparison of CNN-RNN model’s F1-score with recent existing works (**b**).

Figure 7a presents a comparative analysis of the proposed CNN-RNN model against existing research models, including CNN, LSTM, and CNN-LSTM, with a focus on recall. The results clearly indicate that the proposed CNN-RNN, LSTM, and CNN-LSTM models achieve superior performance, each attaining a recall score of 100.00%. This suggests that these models excel at correctly identifying all relevant instances, minimizing false negatives, and ensuring comprehensive detection. In comparison, the CNN model achieves a recall of 99.00%, which is still strong but lower than the other models.

The lower recall of the CNN model can be attributed to its design, which focuses on feature extraction from spatial data. While CNN is highly effective at recognizing patterns and distinguishing between classes, it may be less efficient in capturing subtle or complex patterns important for recall. In tasks in which identifying all relevant instances (true positives) is crucial, CNN might miss some of the less obvious or harder-to-detect cases, leading to a small number of false negatives and thus slightly lowering the recall. On the other hand, models like CNN-RNN, LSTM, and CNN-LSTM are better equipped to handle sequential and temporal dependencies, making them more effective in capturing all relevant information over time. The integration of the RNN or LSTM layers allows these models to not only focus on spatial features but also learn from temporal context, which helps in maintaining a higher recall. As a result, these models can better capture all relevant instances, even those that might be missed by CNN alone, leading to a perfect recall score of 100. CNN’s lower recall is a compromise for its focused, but less context-aware, spatial feature extraction.

Figure 7b presents a comparative analysis of the proposed CNN-RNN model against existing research models, including CNN, LSTM, and CNN-LSTM, focusing specifically on the F1-score. The results clearly indicate that all models of CNN-RNN, LSTM, CNN, and CNN-LSTM can achieve the same F1-score of 99.00%. This suggests that despite the architectural differences between the models, they exhibit similar performance in terms of balancing precision and recall. The F1-score is a way of measuring the accuracy of a system. It takes both false positives and false negatives into consideration. In this case, the fact that all models achieve an F1-score of 99% indicates that they are all very well tuned to strike a balance between precision and recall with only minor differences in their individual metrics. Each model effectively minimizes both false positives and false negatives to a similar extent, ensuring that their precision and recall scores are well aligned. One more reason for all models achieving the same F1-score is because the task or dataset is relatively straightforward, allowing all models to perform well within their respective strengths. For instance, if the data are well structured or the problem is less complex, both CNN (focused on spatial patterns) and LSTM (focused on temporal dependencies) models may handle the features effectively without either precision or recall suffering significantly. Additionally, the CNN-RNN and CNN-LSTM models, which integrate both convolutional and recurrent layers, might also be benefiting from the complementary strengths of these components. CNN excels at feature extraction, while LSTM handles sequential relationships, resulting in an optimal balance between precision and recall that leads to the same F1-score as the other models.

Table 2 presents a comparison of the mean absolute error (MAE) values across different models. The CNN-RNN model achieves the lowest MAE, with a value of 0.0354, indicating that it has the highest prediction accuracy and the smallest average error. Following the CNN-RNN, the CNN-LSTM model shows an MAE of 0.0400, performing better than the other models but still falling slightly behind the CNN-RNN. The LSTM and CNN models have higher MAE values of 0.0420 and 0.0440, respectively. The lower MAE of the CNN-RNN model can be attributed to its hybrid architecture, which effectively combines the advantages of both convolutional and recurrent layers. The convolutional layers excel at extracting complex spatial features, identifying relevant patterns, and reducing noise, while the recurrent layers, like LSTM, handle temporal dependencies and sequential relationships in the data. This combination allows the CNN-RNN to comprehensively capture both spatial and temporal patterns, resulting in more precise predictions and lower errors.

On the other hand, while the CNN-LSTM model also integrates convolutional and recurrent elements, its slightly higher MAE suggests that it may not manage the balance between spatial and temporal features as effectively as the CNN-RNN. This might be due to differences in how these two models’ sequence and process information. CNN-RNN architectures often involve direct interplay between the CNN and RNN components, optimizing the feature learning process more efficiently. For LSTM and CNN models, the higher MAE values indicate their weakness when handling certain types of data. LSTM models are excellent at managing sequential data and temporal dependencies, but they might not perform as well with spatial data without the help of convolutional layers. Meanwhile, CNNs are highly effective in extracting spatial features, but they lack the capacity to consider temporal context, which can lead to slight inaccuracies in predictions when temporal dynamics are involved. In summary, the CNN-RNN’s ability to capture and integrate both spatial and temporal patterns with greater accuracy contributes to its lower MAE, making it more effective for complex data scenarios where both types of features are essential for precise predictions.

**Figure 8:**
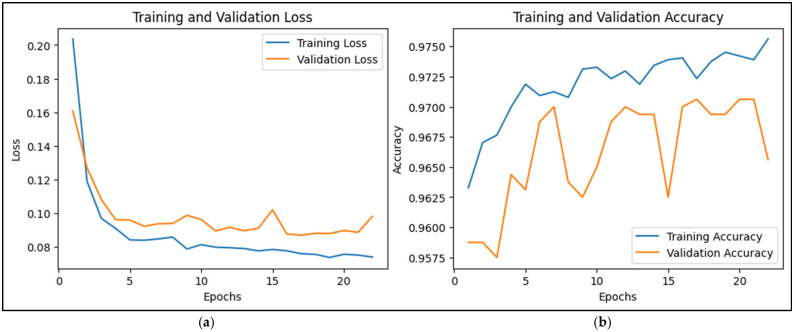
CNN-LSTM-based training and validation loss with 50 epochs (**a**); CNN-LSTM-based training and validation accuracy with 50 epochs (**b**).

The training and validation loss curves in the provided CNN-LSTM training output offer insights into the model’s performance over the 22 epochs, which is shown in Figure 8a. Training loss refers to the error measured on the training dataset after each epoch, indicating how well the model fits the training data. In contrast, validation loss is the error on a separate validation dataset not used during training, serving as an estimate of how well the model generalizes to unseen data or prediction data. According to Figure 8a, the training loss of the CNN-LSTM model started at 0.2082 and decreased steadily to around 0.0728 by the 22nd epoch, while the validation loss began at 0.1609, initially dropped, and then fluctuated around 0.0870 to 0.0982.

During the first few epochs (1 to 6), both training and validation losses decreased, suggesting that the model was learning well and generalizing effectively to the validation set. However, from around epoch 7 onward, the validation loss began to fluctuate, indicating that the model’s capacity to improve generalization on the validation set was stabilizing. Although the training loss continued to decline steadily, the validation loss did not show the same consistent improvement, hinting at potential overfitting. This is evident from the gap between the final training and validation losses. While the training loss kept decreasing, the validation loss did not improve significantly. Around epoch 6, the performance stabilization for validation loss suggests that the model may have reached its optimal learning capacity with the current hyperparameters and training setup. Even though the training loss continued to reduce, indicating fewer errors on the training set, the lack of a corresponding decrease in validation loss implies that the model was learning patterns specific to the training data, potentially at the expense of generalization. This situation often signals the onset of overfitting, where the model is memorizing details rather than learning general patterns.

To address these concerns, several strategies can be considered. Regularization techniques like dropout or L2 regularization could help mitigate overfitting. Implementing early stopping around epochs 12 to 16, when the validation loss stopped improving, could prevent the model from overfitting further. Additionally, increasing the size of the training dataset or employing data augmentation might improve generalization. Adjusting hyperparameters, such as learning rates and the model’s complexity, could also help find a better balance between fitting the training data and generalizing well to validation data. Despite the challenges with validation loss, the model’s accuracy trends were positive. Training accuracy improved from 94.99% to 97.71% over the epochs, and validation accuracy remained high, ranging from 95.75% to 97.06%. This stability in accuracy indicates that while there are minor issues with overfitting, the model’s overall performance on prediction data remains strong. It highlights the fact that a lower loss does not always equate to better accuracy, as the model might still make smaller errors on the training data while misclassifying certain validation samples.

The CNN-LSTM model’s training and validation accuracy patterns, which are shown in Figure 8b, provide valuable understanding into its learning performance. Initially, the training accuracy increased steadily from 94.99% in the first epoch to 97.71% by the 22nd epoch. This consistent improvement indicates that the model was learning patterns in the training data effectively, making fewer classification errors as training progressed. Validation accuracy, which measures the model’s performance on prediction data, started at 95.88% in the first epoch and generally remained high, fluctuating between 95.75% and 97.06% across the epochs. Significantly, the validation accuracy showed only minor changes, suggesting that the model maintained stable performance on the validation set. Peaks in validation accuracy, such as reaching 97.06% in multiple epochs, indicate moments where the model generalized well to new data. The difference between training and validation accuracy highlights how well the model generalized. Training accuracy continued to improve, showing the model was mastering the training data. However, the relatively stable validation accuracy, with occasional slight dips, suggested that after a certain point, the model’s additional learning did not lead to better performance on the validation data. This indicates a potential onset of overfitting, as the model’s capacity to generalize beyond the training data plateaued. In summary, while the model achieved high accuracy on both training and validation sets, the consistent upward trend in training accuracy, contrasted with the stable but fluctuating validation accuracy, suggests a slight overfitting tendency. Adjustments such as early stopping, regularization, or data augmentation could help improve generalization further.

**Figure 9:**
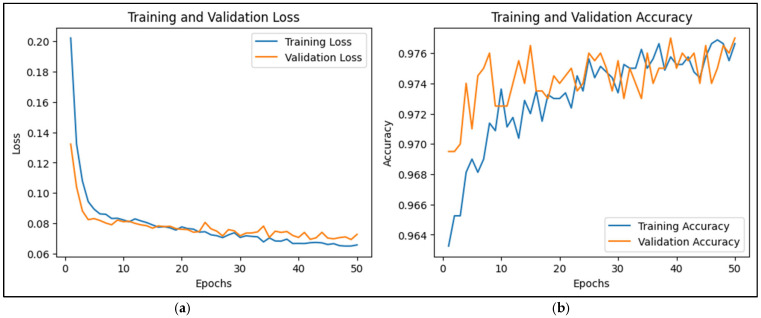
CNN-RNN training and validation loss with 50 epochs (**a**); CNN-RNN training and validation accuracy with 50 epochs (**b**).

The training and validation loss results from your CNN-RNN classifier over 50 epochs, which are shown in Figure 9a, provide insight into the learning dynamics of the model. Initially, during the first few epochs, the model exhibits a noticeable reduction in both training and validation losses. For instance, at epoch 1, the training loss starts at 0.2083 with a validation loss of 0.1375. By epoch 3, the training loss drops to 0.1311 while the validation loss is at 0.1018, indicating the model is quickly learning and improving. As training progresses, both training and validation losses continue to decrease, although at a slower pace. The training loss stabilizes to values ranging between 0.1 and 0.08, and the validation loss consistently decreases as well, maintaining a range of 0.09 to 0.08 by epoch 10. This reduction in loss reflects that the model is finding patterns in the data, leading to a steady improvement in both training and validation accuracy. From epochs 11 to 20, the training loss fluctuates slightly but shows an overall decline. A notable observation is that the validation loss remains relatively stable even achieving lower values compared to earlier epochs. This suggests that the model maintains generalization ability without overfitting, which is evidenced by the validation loss not rising significantly despite minor fluctuations in training loss.

In the later epochs, from 30 onwards, both training and validation losses reach more consistent and lower values with the training loss converging around the 0.06 to 0.07 range. The validation loss similarly stabilizes, hovering between 0.07 and 0.08. This indicates that the model has converged, learning the key patterns in the data while effectively managing to avoid overfitting. The minimal gap between the training and validation losses highlights a good generalization capability of the CNN-RNN model. Overall, the CNN-RNN classifier exhibits a healthy learning pattern. The gradual decrease and stabilization of both training and validation losses, along with consistent validation accuracy improvements, demonstrate that the model effectively learns the underlying features of the data while avoiding overfitting. This is evidenced by the close values of training and validation losses by the end of the 50 epochs.

The training accuracy and validation accuracy of the CNN-RNN classifier, which are shown in Figure 9b, show a clear progression of improvement throughout the 50 epochs. Initially, the training accuracy starts at 94.99% and gradually increases to around 98.00% by the final epoch. The validation accuracy follows a similar upward trend, beginning at 96.95% and reaching 97.75% by epoch 50. The specific relationship between the training and validation accuracy indicates a consistent and stable performance. While both metrics show improvement, there is a small but consistent gap between the two with the validation accuracy typically being slightly higher or at parity with the training accuracy. This could suggest that the model generalizes well to prediction data and is not overfitting, as the validation accuracy remains competitive with the training accuracy across epochs.

In the early stages, the training accuracy increases quickly, whereas the validation accuracy remains relatively stable. Over time, both accuracies become more stable with slight fluctuations observed toward the later epochs. This suggests that the model is learning effectively from the training data while maintaining strong performance on validation data, which is an indication of good generalization of the proposed model. The slight difference between training and validation accuracy in later epochs further supports the model’s ability to generalize without overfitting.

Based on the obtained results, the CNN-RNN model demonstrates superior performance compared to the CNN-LSTM model in terms of both training and validation loss as well as accuracy. Over the 50 epochs, the CNN-RNN model shows a steady and significant increase in training accuracy, reaching 98.00% by the final epoch while maintaining a high validation accuracy of 97.75%. However, the CNN-LSTM model demonstrates lower performance compared to the CNN-RNN model in terms of both training and validation loss as well as accuracy. Over the 50 epochs, the CNN-RNN model shows a steady and significant increase in training accuracy, improving from 94.99% to 98.00% by the final epoch. This shows that this model can learn effectively from the training data and perform well on prediction data with minimal overfitting.

All in all, the proposed CNN-RNN model has a validation accuracy of 97.75% and a training accuracy that improved from 94.99% to 98.00%. The CNN-LSTM model’s training accuracy improved from 94.99% to 97.71% over the epochs, while the validation accuracy ranged from 95.75% to 97.06%. Based on the results, the gaps between these accuracies help identify the strength or accuracy of the proposed model. It is evident that the proposed model not only achieves higher training and validation accuracy than the existing CNN-LSTM model but also has lower training and validation loss compared to current studies using CNN-LSTM. This suggests that the proposed model is better at minimizing error and can generalize more effectively to prediction data. Significantly, based on experimental results, the proposed model’s gap between training and validation accuracy is only 0.25%. In contrast, the gap between training and validation accuracy for the CNN-LSTM model is as high as 0.65%, indicating that the proposed model is more stable and advanced than existing models. This smaller gap also reflects the model’s ability to avoid overfitting, ensuring that it performs consistently across both training and validation datasets.

## 5. Conclusions

In conclusion, the proposed CNN-RNN model offers enhanced reliability in terms of accuracy and stability, making it an ideal solution for predictive maintenance and error detection on motor drives of industrial robots, outperforming other existing research models such as CNN, LSTM, and CNN-LSTM as well as traditional machine learning models. The proposed CNN-RNN can accurately predict and detect the error of motor drives on industrial robots, which is specific to the result metric, such as accuracy, recall, precision, and F1-scores. This approach not only enhances reliability but also saves significant time and effective maintenance, which can improve overall industrial efficiency. Even though the CNN-LSTM model shows steady improvements in accuracy, reaching a final training accuracy of approximately 97.71%, with validation accuracy stabilizing around 96–97%, its performance still stands behind the CNN-RNN model. CNN-RNN validation loss decreases consistently throughout training, achieving a final validation loss of about 0.0688, indicating effective model optimization, which demonstrates significant improvements, with a higher training accuracy of around 98.00% and a validation accuracy peaking at approximately 97.75%. When comparing the two models, both exhibit similar trends in terms of accuracy and loss reduction with CNN-RNN showing a marginally better final performance. Overall, the CNN-RNN model provides more robust performance in terms of accuracy and stability, making it particularly effective for predictive maintenance and fault detection in motor drive control systems of industrial robots. Future work will explore advanced AI techniques, such as transfer and reinforcement learning, for error detection in motor drives of industrial robots. It will also focus on improving scalability and expanding the dataset to enhance model robustness and generalization across various industrial environments.

## Figures and Tables

**Table 1:** The values of parameter settings for the proposed model.

Number	Parameter	Value
1	Conv1D layer	64 filters, kernel size of 3, ReLU activation
2	Dropout rate	20%
3	RNN layer	32 units, no sequence output
4	Dense layer	1 unit with sigmoid activation
5	Optimizer	Adam
6	Loss function	Binary cross-entropy
7	Batch size	32
8	Epochs	50

**Table 2:** Mean absolute error (MAE) comparison by difference models.

Number	Model	Value
1	CNN	0.0440
2	LSTM	0.0420
3	CNN-LSTM	0.0400
4	CNN-RNN	0.0354

## Data Availability

The raw data supporting the conclusions of this article will be made available by the authors on request.
